# Smithsonian Institution—Hodgkins Fund Prize

**Published:** 1908-03

**Authors:** Charles D. Walcott

**Affiliations:** Washington, D. C.


### Smithsonian Institution—Hodgkins Fund Prize

### A Prize of $1,500 Offered for the Best Treatise on the Relation of Atmospheric Air to Tuberculosis

In October, 1891, Thomas George Hodgkins, Esquire, of Setauket, New York, made a donation to the Smithsonian Institution, the income from a part of which was to be devoted to "the increase and diffusion of more exact knowledge in regard to the nature and properties of atmospheric air in connection with the welfare of man." In furtherance of the donor's wishes, the Smithsonian Institution has from time to time offered prizes, awarded medals, made grants for investigations, and issued publications.

In connection with the approaching International Congress on Tuberculosis, which will be held in Washington, September 21 to October 12, 1908, a prize of $1,500 is offered for the best treatise "On the Relation of Atmospheric Air to Tuberculosis." Memoirs having relation to the cause, spread, prevention, or cure of tuberculosis are included within the general terms of the subject.

Any memoir read before the International Congress on Tuberculosis, or sent to the Smithsonian Institution or to the Secretary-General of the Congress before its close, namely, October 12, 1908, will be considered in the competition.

The memoirs may be written in English, French, German, Spanish, or Italian. They should be submitted either in manuscript or typewritten copy, or if in type, printed as manuscript. If written in German, they should be in Latin script. They will be examined and the prize awarded by a Committee appointed by the Secretary of the Smithsonian Institution in conjunction with the officers of the International Congress on Tuberculosis.

Such memoirs must not have been published prior to the Congress. The Smithsonian Institution reserves the right to publish the treatise to which the prize is awarded. No condition as to the length of the treatises is established, it being expected that the practical results of important investigations will be set forth as convincingly and tersely as the subject will permit. The right is reserved to award no prize if in the judgment of the Committee no contribution is offered of sufficient merit to warrant such action.

Memoirs designed for consideration should be addressed to either "The Smithsonian Institution, Washington, District of Columbia, U. S. A.;" or to "Dr. John S. Fulton, Secretary-General of the International Congress on Tuberculosis, 714 Colorado Building, Washington, District of Columbia, U. S. A." Further information, if desired by persons intending to become competitors, will be furnished on application.

CHARLES D. WALCOTT,
*Secretary of the Smithsonian Institution.*

WASHINGTON, D. C.,
February 3, 1908.

